# Supplementary material for: Comparison of the Transcriptomes and Proteomes of Serum Exosomes from Marek’s Disease Virus-Vaccinated and Protected and Lymphoma-Bearing Chickens
Source: Genes (Basel). 2019 Feb 5;10(2):116. doi: 10.3390/genes10020116 (PMC6410298; doi:10.3390/genes10020116)
Supplement: Supplementary file 1 [file genes-10-00116-s001.zip › Supplementary Data/Supplementary Figures.pptx]

## Slide 1
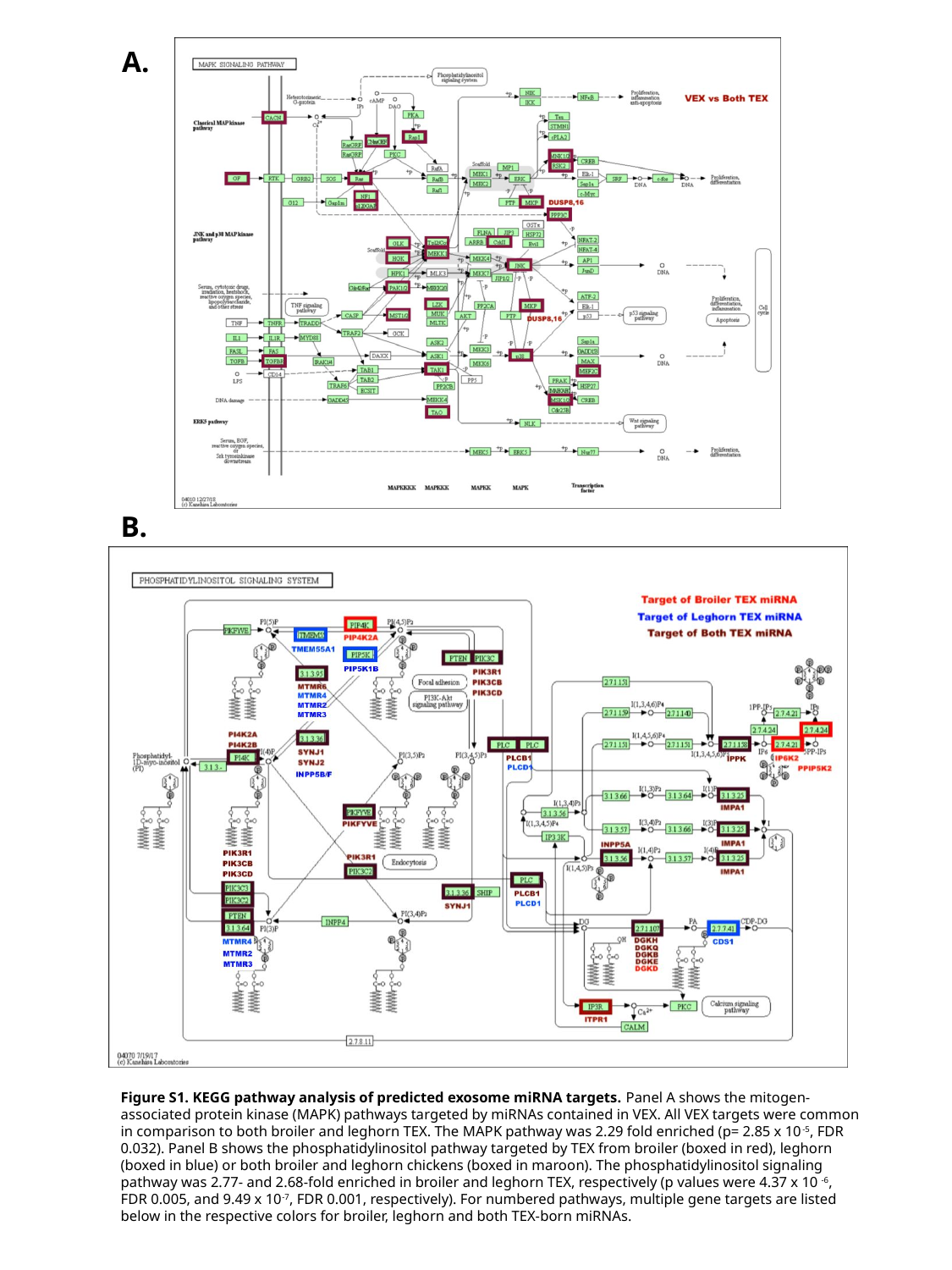

A.
B.
Figure S1. KEGG pathway analysis of predicted exosome miRNA targets. Panel A shows the mitogen-associated protein kinase (MAPK) pathways targeted by miRNAs contained in VEX. All VEX targets were common in comparison to both broiler and leghorn TEX. The MAPK pathway was 2.29 fold enriched (p= 2.85 x 10^-5^, FDR 0.032). Panel B shows the phosphatidylinositol pathway targeted by TEX from broiler (boxed in red), leghorn (boxed in blue) or both broiler and leghorn chickens (boxed in maroon). The phosphatidylinositol signaling pathway was 2.77- and 2.68-fold enriched in broiler and leghorn TEX, respectively (p values were 4.37 x 10 -6, FDR 0.005, and 9.49 x 10^-7^, FDR 0.001, respectively). For numbered pathways, multiple gene targets are listed below in the respective colors for broiler, leghorn and both TEX-born miRNAs.

## Slide 2
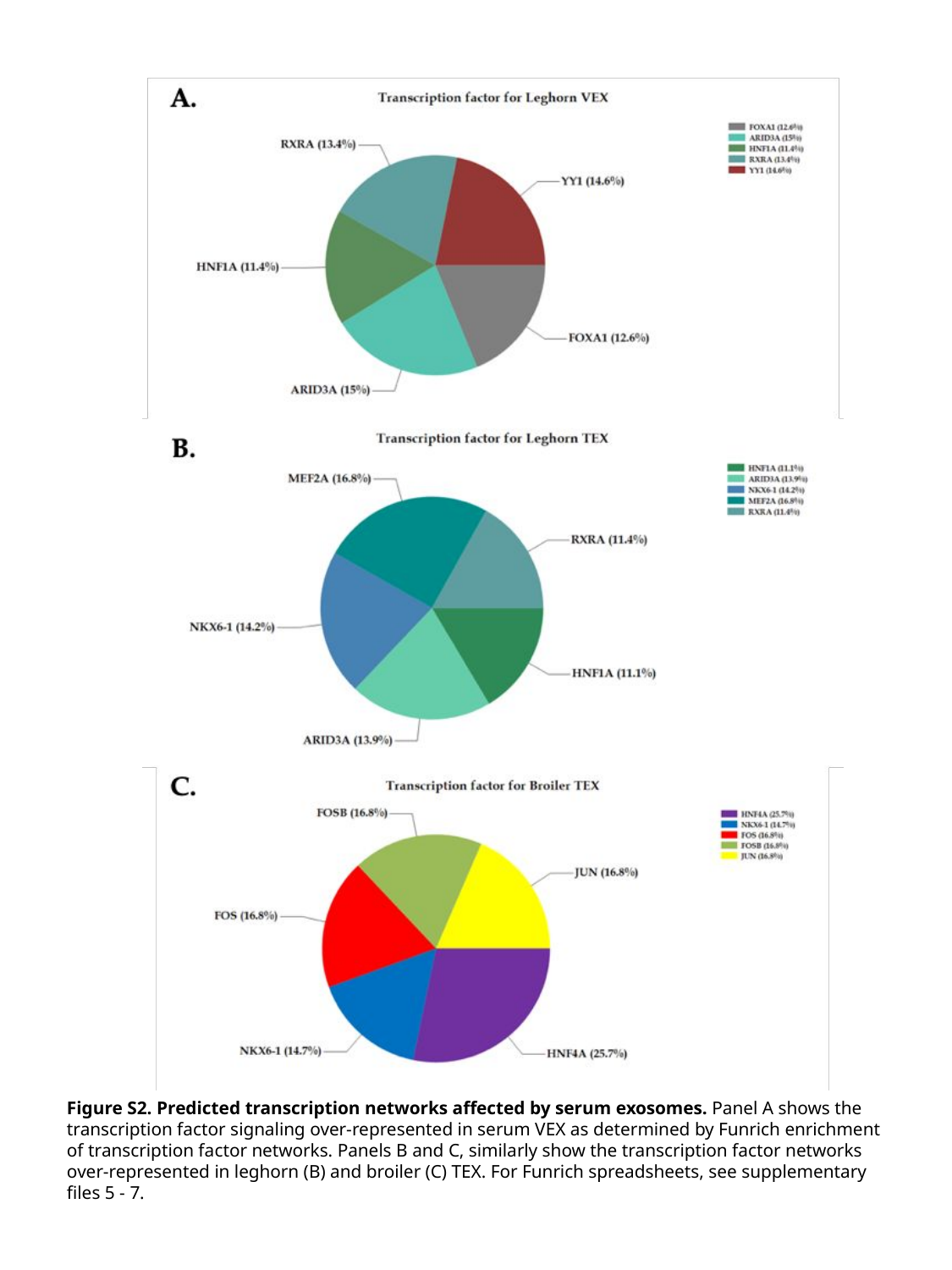

Figure S2. Predicted transcription networks affected by serum exosomes. Panel A shows the transcription factor signaling over-represented in serum VEX as determined by Funrich enrichment of transcription factor networks. Panels B and C, similarly show the transcription factor networks over-represented in leghorn (B) and broiler (C) TEX. For Funrich spreadsheets, see supplementary files 5 - 7.
