# Supplementary material for: Comparison of the Transcriptomes and Proteomes of Serum Exosomes from Marek’s Disease Virus-Vaccinated and Protected and Lymphoma-Bearing Chickens
Source: Genes (Basel). 2019 Feb 5;10(2):116. doi: 10.3390/genes10020116 (PMC6410298; doi:10.3390/genes10020116)
Supplement: Supplementary file 1 [file genes-10-00116-s001.zip › Supplementary Data/Supplementary Tables-revised.docx]

**SUPPLEMENTAL TABLES**

**Table S1. MDV-1 miRNA read counts for the 12 libraries**

|  | **MDV1-miR-** | | | | | | | | | | | | | |
| --- | --- | --- | --- | --- | --- | --- | --- | --- | --- | --- | --- | --- | --- | --- |
|  | ***meq* cluster** | | | | | | | | | **LAT cluster** | | | | |
|  | **Cluster 1** | | | | | | **Cluster 2** | | |  |  |  |  |  |
| **Bird tag#** | **-M9** | **-M5** | **-M12** | **-M3** | **-M2** | **-M4** | **-M11** | **-M31** | **-M1** | **-M8** | **-M13** | **-M6** | **-M7** | **-M10** |
| BL3815^a^ | 6 | 3 | 75 | 54 | 14 | 226 | 1 | 0 | 1 | 31 | 0 | 42 | 6 | 19 |
| BL3841^a^ | 9 | 0 | 83 | 21 | 10 | 74 | 0 | 0 | 0 | 7 | 0 | 12 | 4 | 4 |
| BL3850^a^ | 155 | 70 | 2,143 | 378 | 327 | 2,555 | 14 | 0 | 39 | 749 | 4 | 346 | 16 | 99 |
| BL3825^a^ | 5 | 40 | 469 | 48 | 109 | 827 | 0 | 0 | 1 | 208 | 5 | 124 | 3 | 25 |
| OR1810^b^ | 1,084 | 63 | 6,400 | 4,788 | 464 | 30,520 | 30 | 0 | 27 | 1,089 | 26 | 2,863 | 391 | 148 |
| OR1760^b^ | 102 | 19 | 653 | 377 | 81 | 1,747 | 3 | 0 | 8 | 686 | 10 | 322 | 48 | 70 |
| OR1838^b^ | 316 | 41 | 1,240 | 465 | 188 | 315 | 16 | 5 | 2 | 1,226 | 2 | 493 | 45 | 45 |
| OR748^b^ | 144 | 43 | 1,248 | 905 | 275 | 5,568 | 15 | 4 | 12 | 834 | 1 | 320 | 167 | 58 |
| BL4178^c^ | 378 | 194 | 6,243 | 833 | 1,563 | 10,271 | 194 | 9 | 106 | 738 | 1 | 1,494 | 144 | 365 |
| BL4047^c^ | 2,307 | 559 | 3,6417 | 5,087 | 5,103 | 26,344 | 221 | 18 | 330 | 4,441 | 31 | 6,329 | 313 | 1,540 |
| BL4062^c^ | 3,109 | 378 | 6,7752 | 10,561 | 7,329 | 42,457 | 369 | 58 | 215 | 6,113 | 33 | 8,801 | 349 | 487 |
| BL4183^c^ | 1,106 | 619 | 25,329 | 5,413 | 4,683 | 27,671 | 254 | 30 | 132 | 2.470 | 32 | 4,941 | 278 | 1,672 |
| **Total number** | 8,721 | 2,029 | 148,052 | 28,930 | 20,146 | 148,575 | 1,117 | 124 | 873 | 18,592 | 145 | 26,087 | 1,764 | 4,532 |

1. Leghorn VEX
2. Leghorn TEX
3. Broiler TEX

**Table S2. MDV-1 *meq* cluster miRNA read proportions in VEX and TEX**

|  | ***meq* cluster (MDV1-miR-)** | | | | | | | | | | **Total**  **number** |
| --- | --- | --- | --- | --- | --- | --- | --- | --- | --- | --- | --- |
|  | **Cluster 1** | | | | |  | **Cluster 2** | | | |  |
| **Sample library** | **-M9** | **-M5** | **-M12** | **-M3** | **-M2** |  | **-M4** | **-M11** | **-M31** | **-M1** |  |
| **leghorn VEX (n=4)** | 175 (2.26%) | 113 (1.46%) | 2770 (35.71%) | 501 (6.46%) | 460 (5.93%) |  | 3682 (47.47%) | 15 (0.19%) | 0 (0%) | 41 (0.53%) | 7757 |
| **leghorn TEX (n=4)** | 1646 (2.88%) | 166 (0.29%) | 9541 (16.69%) | 6535 (11.43%) | 1008 (1.76%) |  | 38150 (66.73%) | 64 (0.11%) | 9 (0.02%) | 49 (0.09%) | 57168 |
| **broiler TEX (n=4)** | 6900 (2.35%) | 1750 (0.6%) | 135741 (46.23%) | 21894 (7.46%) | 18678 (6.36%) |  | 106743 (36.35%) | 1038 (0.35%) | 115 (0.04%) | 783 (0.27%) | 293642 |

**Table S3. MDV-1 LAT cluster miRNA read proportions in VEX and TEX**

|  | **LAT cluster (MDV1-miR-)** | | | | | **Total number** |
| --- | --- | --- | --- | --- | --- | --- |
|  |  |  |  |  |  |  |
| **Sample library** | **-M8** | **-M13** | **-M6** | **-M7** | **-M10** |  |
| **leghorn VEX (n=4)** | 995 (58.39%) | 9 (0.53%) | 524 (30.75%) | 29 (1.7%) | 147 (8.63%) | 1704 |
| **leghorn TEX (n=4)** | 3835 (43.36%) | 39 (0.44%) | 3998 (45.21%) | 651 (7.36%) | 321 (3.63%) | 8844 |
| **broiler TEX (n=4)** | 13762 (33.92%) | 97 (0.24%) | 21565 (53.15%) | 1084 (2.67%) | 4064 (10.02%) | 40572 |

**Table S4. miRDB-predicted gene targets for select VEX miRNAs and gene targets**

| **VEX-upregulated miRNA** | **predicted gene targets** | **gene target score ≥ 80** |
| --- | --- | --- |
| gga-miR-146 (-a^1^, -b^1,2^, -c^1,2^) | 129 | 31 |
| gga-miR-143^1^ | 375 | 108 |
| gga-miR-10b^1,2^ | 117 | 30 |
| gga-miR-1288^1,2^ | 79 | 12 |
| gga-miR-27b^1,2^ | 537 | 201 |
| gga-miR-99a^1,2^ | 18 | 8 |
| gga-miR-26a^1,2^ | 345 | 130 |
| let-7f^2^ | 204 | 81 |
| gga-miR-24^1^ | 265 | 70 |
| gga-miR-23b^1^ | 499 | 200 |
| gga-miR-1388^1,2^ | 89 | 36 |
| gga-miR-30e^2^ | 526 | 241 |

1. Selected miRNA upregulated in leghorn VEX compared to leghorn TEX
2. Selected miRNA upregulated in leghorn VEX compared to broiler TEX

**Table S5. miRDB-predicted gene targets for select TEX miRNAs and gene targets**

| **TEX-upregulated miRNA** | **predicted gene targets** | **gene target score ≥ 80** |
| --- | --- | --- |
| gga-miR-16c^1^ | 982 | 363 |
| gga-miR-16^1^ | 987 | 364 |
| gga-miR-15c^1^ | 985 | 358 |
| gga-miR-140^2^ | 313 | 67 |
| gga-miR-7^1^ | 227 | 75 |
| gga-miR-142^1,2^ | 277 | 98 |
| gga-miR-21^1,2^ | 119 | 36 |
| gga-miR-148a^1,2^ | 570 | 214 |
| gga-miR-126^2^ | 6 | 0 |
| gga-miR-181a^1,2^ | 526 | 196 |
| gga-miR-451^1^ | 12 | 1 |
| let-7g^1^ | 206 | 88 |
| let-7i^1,2^ | 207 | 88 |
| gga-miR-101^1^ | 645 | 240 |
| gga-miR-125b^1^ | 190 | 66 |
| gga-miR-425^2^ | 129 | 25 |
| gga-miR-92^1,2^ | 355 | 139 |
| gga-miR-363^1,2^ | 367 | 154 |
| gga-miR-122^2^ | 103 | 26 |
| gga-miR-199^2^ | 213 | 65 |
| MDV1-miR-M9^1,2^ | 14 | 0 |
| MDV1-miR-M5^2^ | 63 | 7 |
| MDV1-miR-M12^1,2^ | 117 | 36 |
| MDV1-miR-M3^1,2^ | 601 | 210 |
| MDV1-miR-M2^1,2^ | 135 | 25 |
| MDV1-miR-M4^1,2^ | 195 | 66 |
| MDV1-miR-M11^1,2^ | 888 | 355 |
| MDV1-miR-M1^2^ | 127 | 68 |
| MDV1-miR-M8^1,2^ | 112 | 33 |
| MDV1-miR-M13^1,2^ | 304 | 77 |
| MDV1-miR-M6^1,2^ | 158 | 31 |
| MDV1-miR-M7^1,2^ | 10 | 10 |

1. Selected miRNA upregulated in leghorn TEX compared to leghorn VEX

Selected miRNA upregulated in broiler TEX compared to leghorn TEX
