# Supplementary material for: Comparison of the Transcriptomes and Proteomes of Serum Exosomes from Marek’s Disease Virus-Vaccinated and Protected and Lymphoma-Bearing Chickens
Source: Genes (Basel). 2019 Feb 5;10(2):116. doi: 10.3390/genes10020116 (PMC6410298; doi:10.3390/genes10020116)
Supplement: Supplementary file 1 [file genes-10-00116-s001.zip › Supplementary Data/Exosome Figures-hi-res-revised.pptx]

## Slide 1
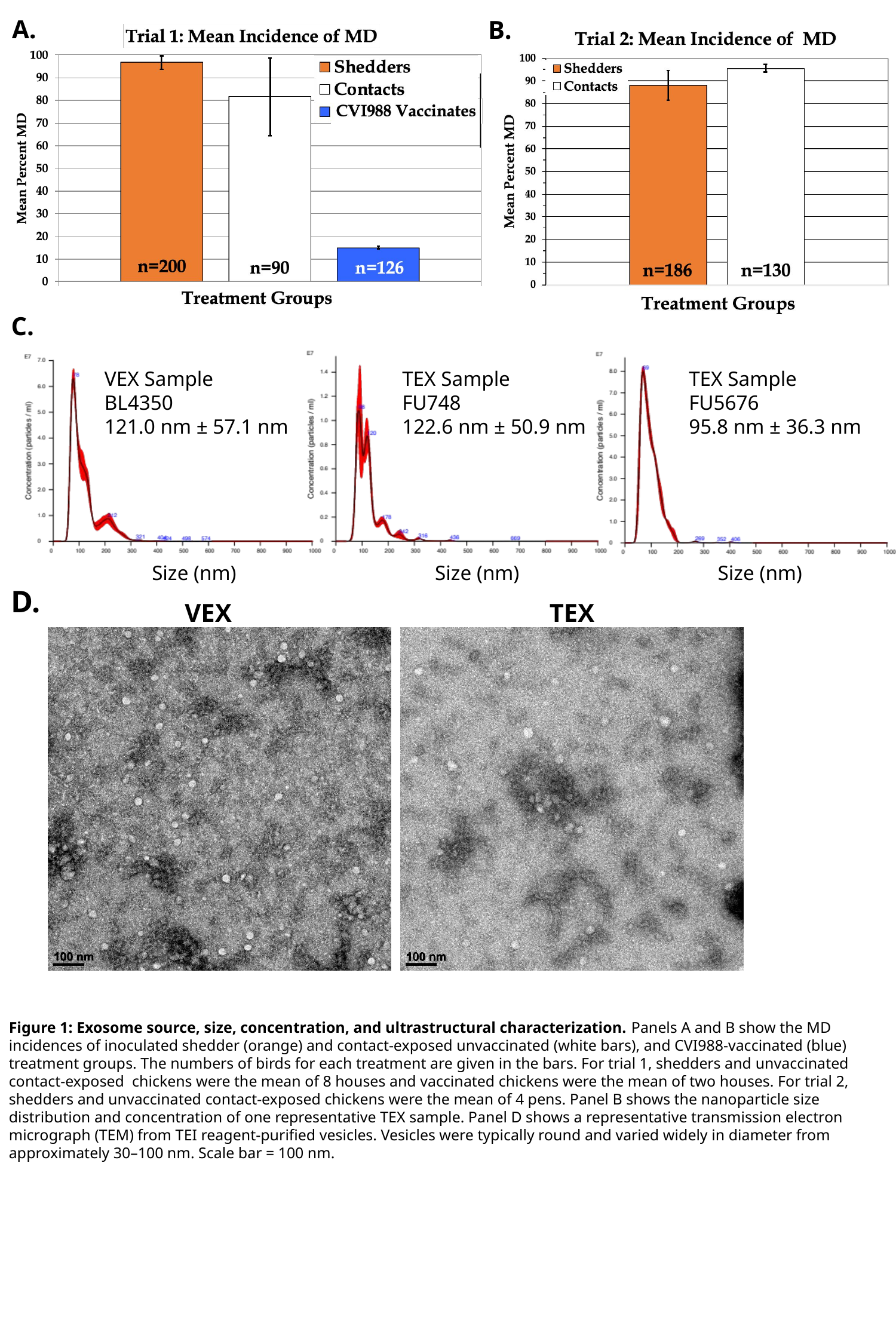

A.
B.
C.
VEX Sample
BL4350
121.0 nm ± 57.1 nm
TEX Sample
FU748
122.6 nm ± 50.9 nm
TEX Sample
FU5676
95.8 nm ± 36.3 nm
Size (nm)
Size (nm)
Size (nm)
D.
VEX
TEX
Figure 1: Exosome source, size, concentration, and ultrastructural characterization. Panels A and B show the MD incidences of inoculated shedder (orange) and contact-exposed unvaccinated (white bars), and CVI988-vaccinated (blue) treatment groups. The numbers of birds for each treatment are given in the bars. For trial 1, shedders and unvaccinated contact-exposed chickens were the mean of 8 houses and vaccinated chickens were the mean of two houses. For trial 2, shedders and unvaccinated contact-exposed chickens were the mean of 4 pens. Panel B shows the nanoparticle size distribution and concentration of one representative TEX sample. Panel D shows a representative transmission electron micrograph (TEM) from TEI reagent-purified vesicles. Vesicles were typically round and varied widely in diameter from approximately 30–100 nm. Scale bar = 100 nm.

## Slide 2
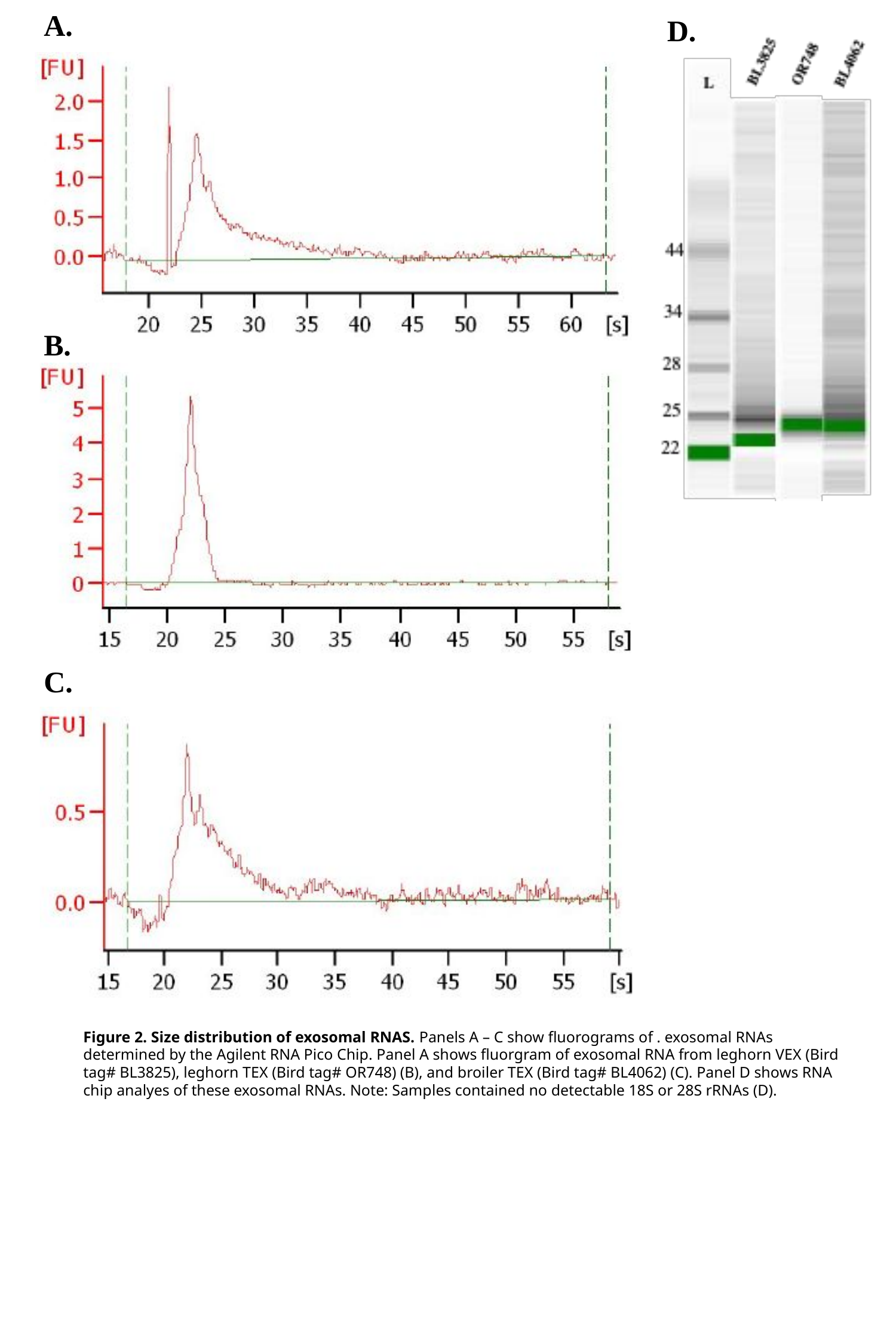

A.
D.
B.
C.
Figure 2. Size distribution of exosomal RNAS. Panels A – C show fluorograms of . exosomal RNAs determined by the Agilent RNA Pico Chip. Panel A shows fluorgram of exosomal RNA from leghorn VEX (Bird tag# BL3825), leghorn TEX (Bird tag# OR748) (B), and broiler TEX (Bird tag# BL4062) (C). Panel D shows RNA chip analyes of these exosomal RNAs. Note: Samples contained no detectable 18S or 28S rRNAs (D).

## Slide 3
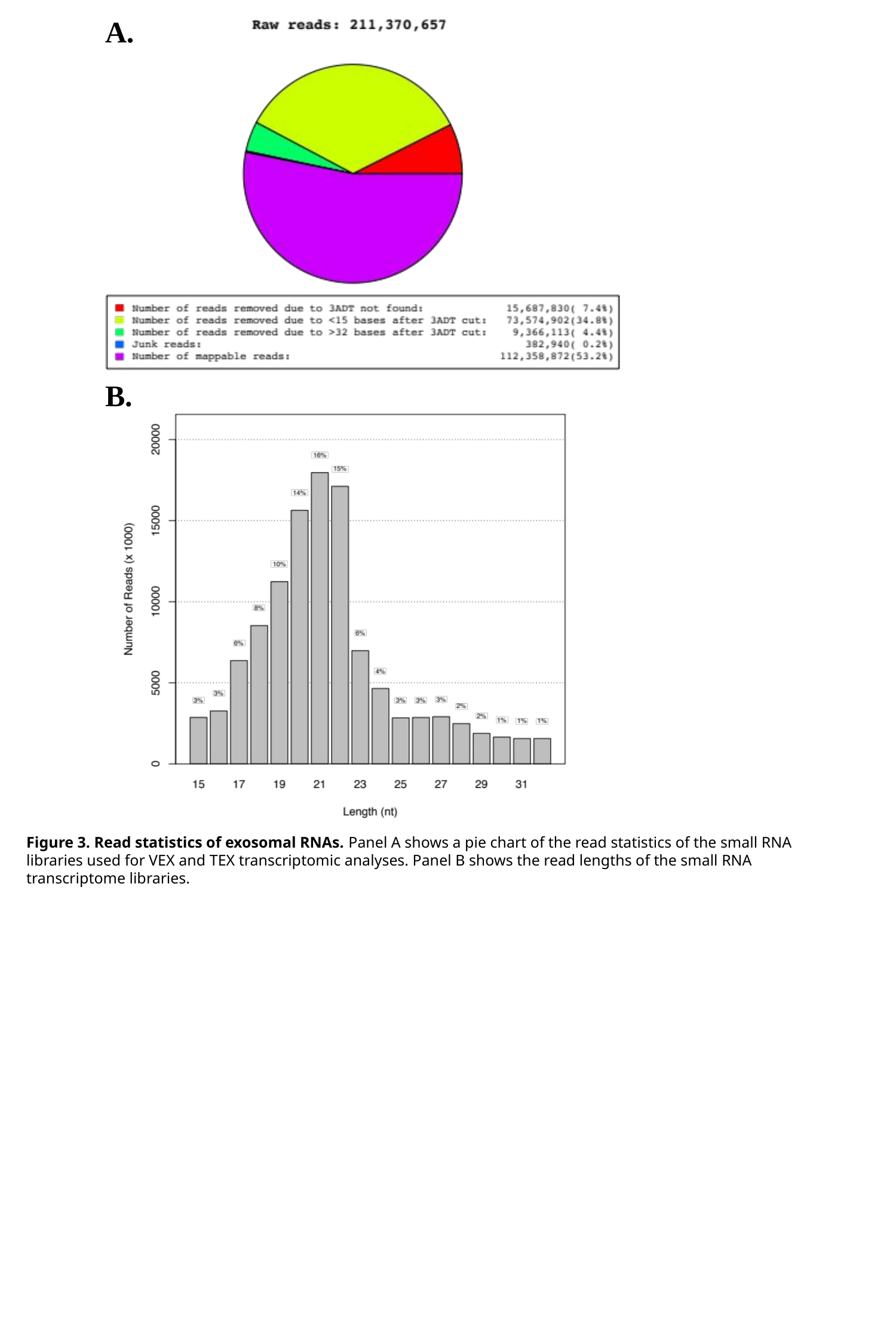

A.
B.
Figure 3. Read statistics of exosomal RNAs. Panel A shows a pie chart of the read statistics of the small RNA libraries used for VEX and TEX transcriptomic analyses. Panel B shows the read lengths of the small RNA transcriptome libraries.

## Slide 4
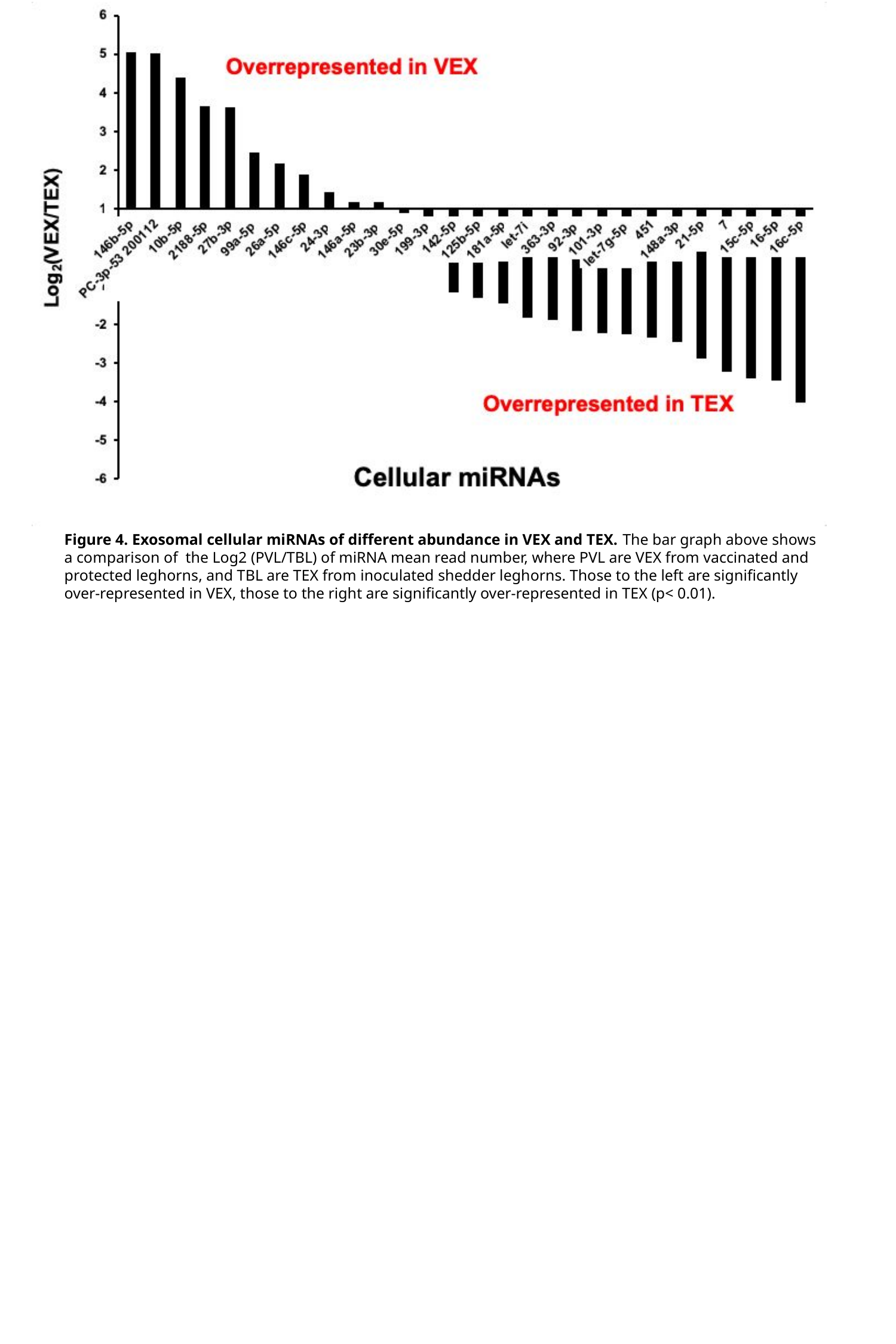

Figure 4. Exosomal cellular miRNAs of different abundance in VEX and TEX. The bar graph above shows a comparison of the Log2 (PVL/TBL) of miRNA mean read number, where PVL are VEX from vaccinated and protected leghorns, and TBL are TEX from inoculated shedder leghorns. Those to the left are significantly over-represented in VEX, those to the right are significantly over-represented in TEX (p< 0.01).

## Slide 5
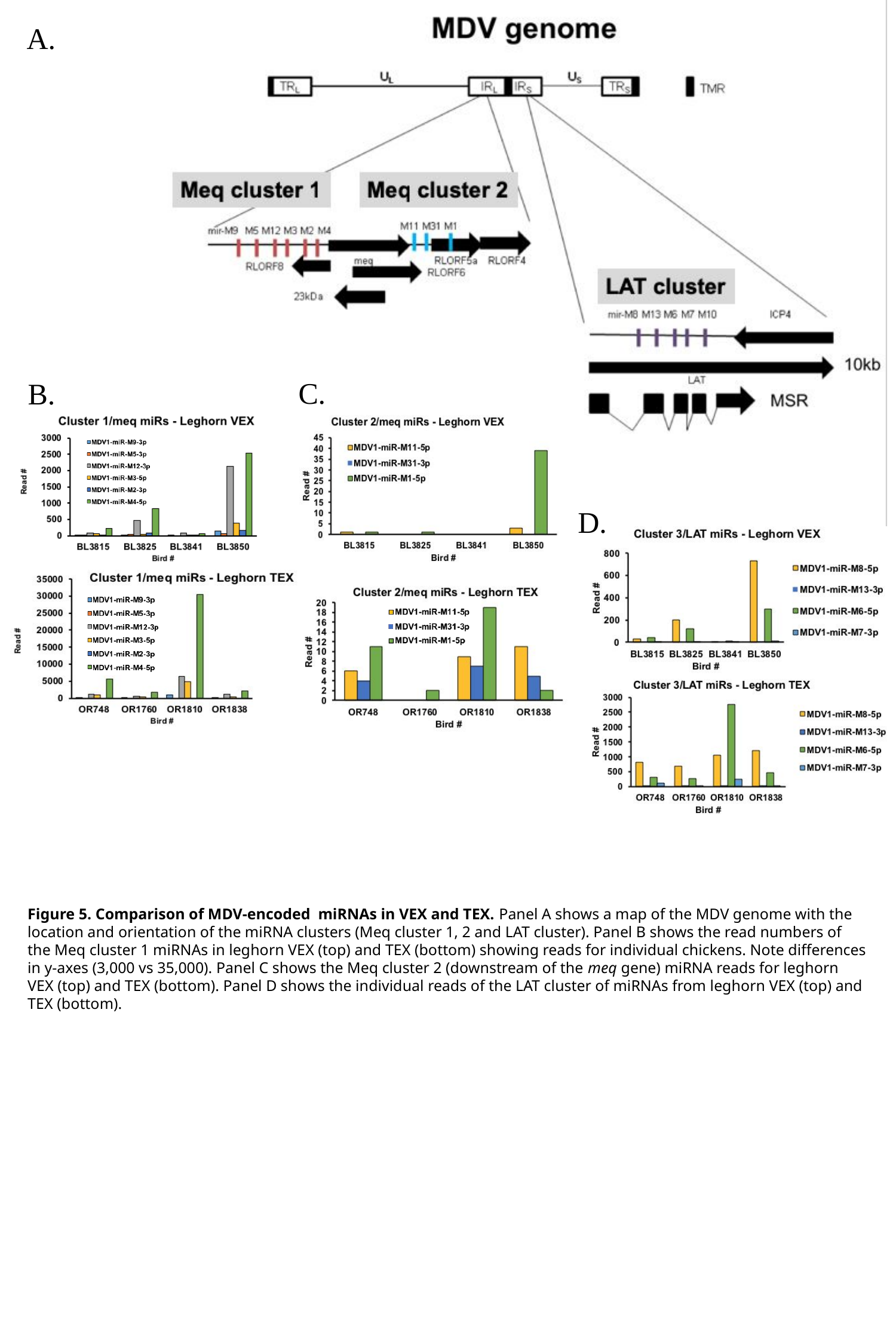

A.
C.
B.
D.
Figure 5. Comparison of MDV-encoded miRNAs in VEX and TEX. Panel A shows a map of the MDV genome with the location and orientation of the miRNA clusters (Meq cluster 1, 2 and LAT cluster). Panel B shows the read numbers of the Meq cluster 1 miRNAs in leghorn VEX (top) and TEX (bottom) showing reads for individual chickens. Note differences in y-axes (3,000 vs 35,000). Panel C shows the Meq cluster 2 (downstream of the meq gene) miRNA reads for leghorn VEX (top) and TEX (bottom). Panel D shows the individual reads of the LAT cluster of miRNAs from leghorn VEX (top) and TEX (bottom).

## Slide 6
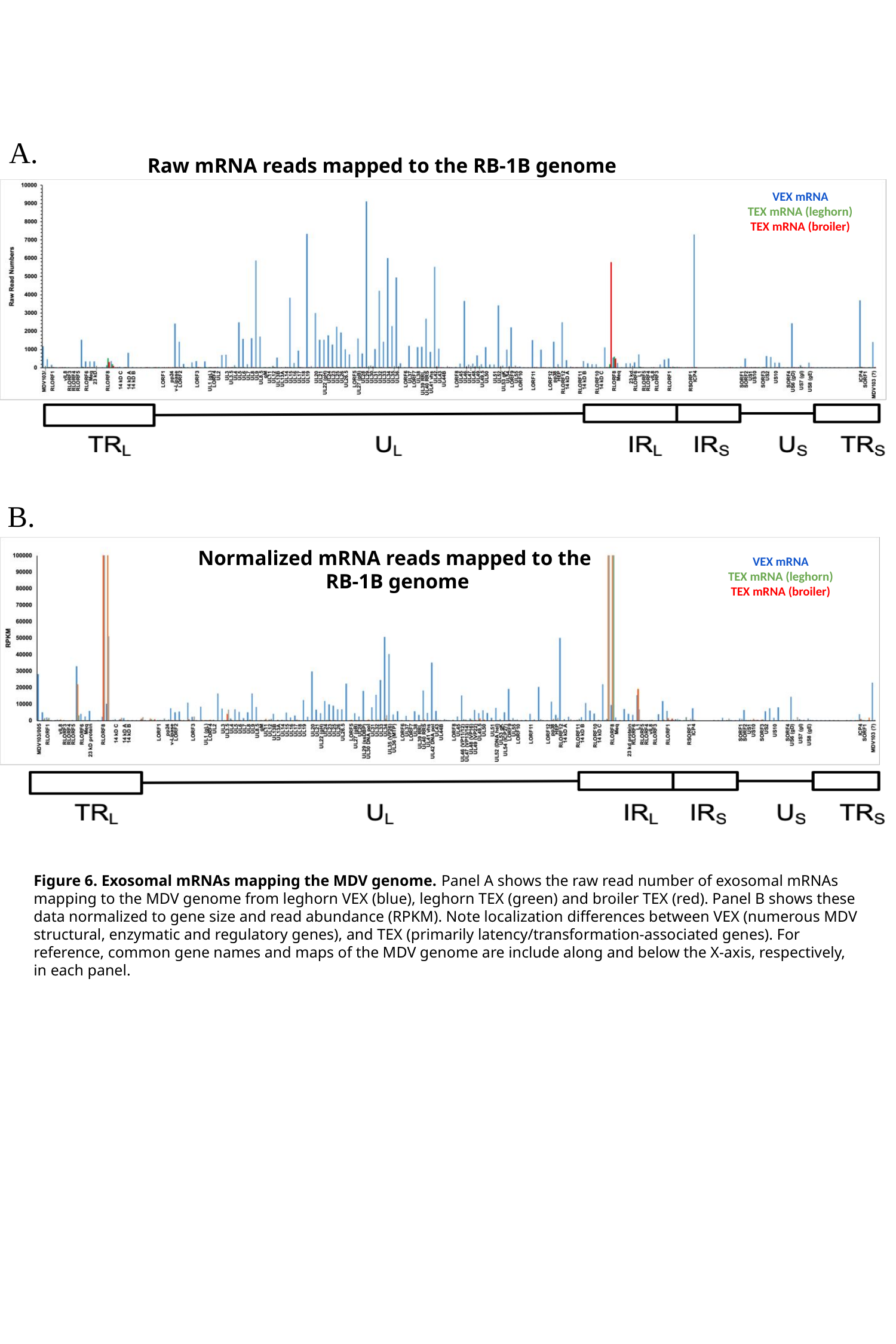

A.
Raw mRNA reads mapped to the RB-1B genome
VEX mRNA
TEX mRNA (leghorn)
TEX mRNA (broiler)
B.
Normalized mRNA reads mapped to the
RB-1B genome
VEX mRNA
TEX mRNA (leghorn)
TEX mRNA (broiler)
Figure 6. Exosomal mRNAs mapping the MDV genome. Panel A shows the raw read number of exosomal mRNAs mapping to the MDV genome from leghorn VEX (blue), leghorn TEX (green) and broiler TEX (red). Panel B shows these data normalized to gene size and read abundance (RPKM). Note localization differences between VEX (numerous MDV structural, enzymatic and regulatory genes), and TEX (primarily latency/transformation-associated genes). For reference, common gene names and maps of the MDV genome are include along and below the X-axis, respectively, in each panel.

## Slide 7
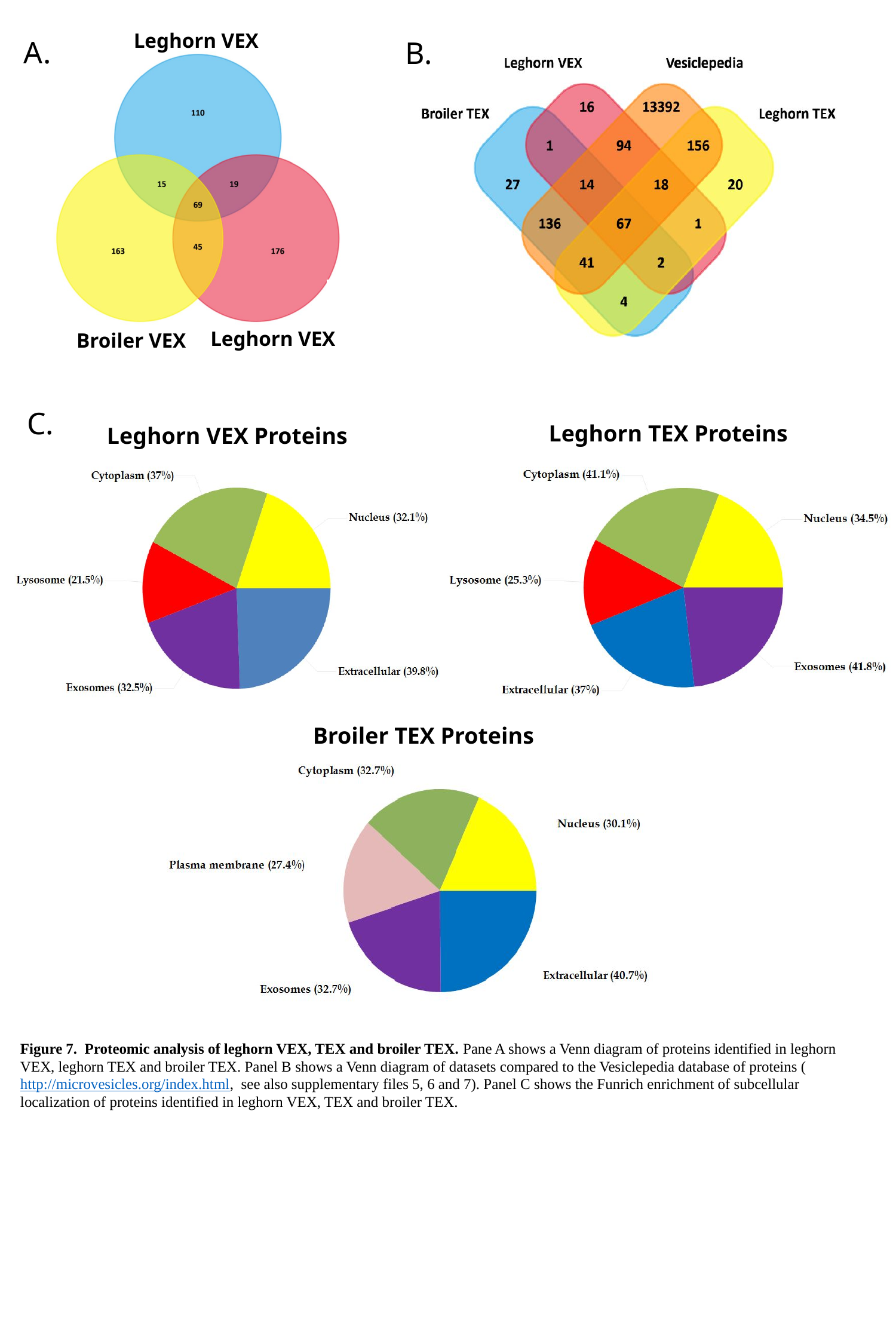

Leghorn VEX
A.
B.
Leghorn VEX
Broiler VEX
C.
Leghorn TEX Proteins
Leghorn VEX Proteins
Broiler TEX Proteins
Figure 7. Proteomic analysis of leghorn VEX, TEX and broiler TEX. Pane A shows a Venn diagram of proteins identified in leghorn VEX, leghorn TEX and broiler TEX. Panel B shows a Venn diagram of datasets compared to the Vesiclepedia database of proteins (http://microvesicles.org/index.html, see also supplementary files 5, 6 and 7). Panel C shows the Funrich enrichment of subcellular localization of proteins identified in leghorn VEX, TEX and broiler TEX.
